# YouTube™ Videos as a Source of Information on Necrotizing Gingivitis: A Content-Quality Analysis

**DOI:** 10.7759/cureus.61485

**Published:** 2024-06-01

**Authors:** Şeyma Çardakcı Bahar, Oğuz Koca

**Affiliations:** 1 Periodontology, Gulhane Faculty of Dentistry, University of Health Sciences, Ankara, TUR

**Keywords:** youtube, necrotizing gingivitis, e-health, dentistry, content analysis

## Abstract

Background

This study aims to evaluate the quality, reliability, and content of the information provided in YouTube™ videos on necrotizing gingivitis (NG), demonstrate the effectiveness of the videos for patients, and help dentists use this platform as a resource to properly guide their patients.

Methodology

This cross-sectional study was conducted by two experienced periodontologists. They began the study by searching for YouTube™ videos using the keywords "necrotizing gingivitis" and "trench mouth." Descriptive parameters such as the source of upload, country of origin, duration, upload date, number of likes, dislikes, views, and comments were evaluated. After this initial evaluation, the viewing rate and interaction index were calculated. Videos were categorized into high content (HC) and low content (LC) based on a 7-point scale. The quality of video content was assessed using the Global Quality Scale (GQS) and the Video Information and Quality Index (VIQI). The data were analyzed using various statistical tests, with a significance level set at p<0.05.

Results

Initially, 148 videos were screened, and 50 videos on NG that met the inclusion criteria were included in the study. Out of the 50 videos, 28 (56%) were uploaded by healthcare professionals. Overall, 68% of videos (n=34) were classified as HC and 32% (n=16) as LC. The most common topic was clinical symptoms and signs of NG, with 86% (n=43), while NG prevention was the least common topic, with 26% (n=13). Statistically significant differences were found between video duration, time since upload, and VIQI scores according to TCS scores (p<0.05). Video duration and VIQI scores were higher for HC videos compared to LC videos. The time since upload for LC videos was higher than for HC videos. Positive correlations were observed between TCS scores, video duration, and VIQI, as well as between GQS scores, video duration, viewing rate, and VIQI.

Conclusions

The majority of NG videos on YouTube™ are useful and comprehensive, but their number is insufficient. Poor-quality and inadequate videos may mislead practitioners and patients. However, this also presents an opportunity for healthcare professionals to leverage YouTube™ as an educational tool. Periodontologists should upload more comprehensive videos and play a more active role in providing high-quality information.

## Introduction

Necrotizing gingivitis (NG) is a severe inflammatory periodontal disease characterized by severe pain, bleeding in the gingiva, and staple hole-like lesions in the interdental papilla [1]. The etiology of NG includes malnutrition, emotional stress, a lack of oral hygiene, systemic diseases, poor sleep patterns, chemotherapy, HIV infection, smoking, and alcohol. NG usually occurs in immunosuppressed states in anaerobic subgingival flora with opportunistic bacteria such as fusiform bacteria and spirochetes [2].

High rates of NG were reported during the Second World War [3], but studies have shown a decrease in prevalence after the war [4,5]. On the other hand, the prevalence of NG varies widely depending on the population but is generally relatively low (<1%) in the industrialized societies of Japan, Europe, and North America [6,7]. This is most likely due to these countries' high standards of health. Interestingly, the true prevalence of NG needs to be further investigated because relevant epidemiological data are often derived from unrepresentative population groups (HIV patients, low socioeconomic class, military officers, soldiers, and urban slum dwellers), possibly providing a skewed estimate [8]. NG is frequently seen in young people between 20 and 25 years old and significantly affects the anterior maxillary and mandibular gingiva [5]. If left untreated, it can progress to necrotizing ulcerative periodontitis due to intraseptal sequestration of alveolar bone, which can spread the disease to surrounding soft tissues, leading to noma or cancrum oris, which can often be fatal [9].

In today's digital society, the Internet provides easy access to health information. According to a study, in the early 2000s, 4.5% of Internet users [10] were looking for health-related information online, while this rate has increased to 80% today [11]. YouTube™, one of the largest video-sharing websites, has approximately two billion users. Compared to other social media platforms, YouTube™ users visit the site nine times a day on average, making it the second most visited website worldwide [12]. It is widely preferred for accessing information because it offers visual and audio content and free access [13].

YouTube™ provides access to many medical topics [11]. It has been reported that it may disseminate misleading and inadequate information because it is a public platform; videos are not scored and do not go through a qualified evaluation system before being uploaded [14]. This study aimed to evaluate the quality and content of the information provided by YouTube™ videos on NG, demonstrate the effectiveness of the videos for patients, and assist dentists in using this platform as a resource to properly guide their patients.

## Materials and methods

Study design and search protocols

To address the purpose of the research, a cross-sectional study was designed. An ethics committee approval for the present study was not required because the source of the study was freely accessible Internet data. At the beginning of the study, a search in Google Trends (Google Trends, 2020, Alphabet, USA) was conducted to determine the most searched keyword related to NG. Google Trends is an online search engine used to determine how often selected key terms are used in queries in a given time period. Search categories were adjusted to ‘‘worldwide,’’ ‘‘last 5 years,’’ and then possible keywords including ''necrotizing gingivitis,'' ''trench mouth,'' ''acute necrotizing gingivitis,'' and ''acute necrotizing ulcerative gingivitis'' were compared. ‘'Necrotizing gingivitis’’ was defined as the most searched keyword, followed by "trench mouth." To broaden the search results, the following two search terms were used as the keywords of the study: (1) necrotizing gingivitis and (2) trench mouth.

Data collection

The study began by activating Google Chrome's incognito mode feature and creating a new YouTube™ user account to ensure that the data collected was accurate and unbiased, without being influenced by previous search history or saved videos. The search was conducted on March 11, 2024, from 7:00 a.m. to 9:00 p.m. No additional filtering options were consciously selected; only the default "relevance" criterion provided by YouTube™ was used. This approach was taken to align the search process with common user practices and replicate the typical search pattern that users follow.

In this study, search results were limited to the first 74 videos for each keyword. Subsequent videos were also initially checked but were not included in the sample as many irrelevant videos were found. A total of 148 videos were saved to a playlist called “NG” in the library section of YouTube™. The URLs of all scanned videos were backed up in a Microsoft Excel file (Microsoft® Corp., Redmond, WA, USA) as the search results may change on different days.

Video selection and evaluation

**Figure 1:**
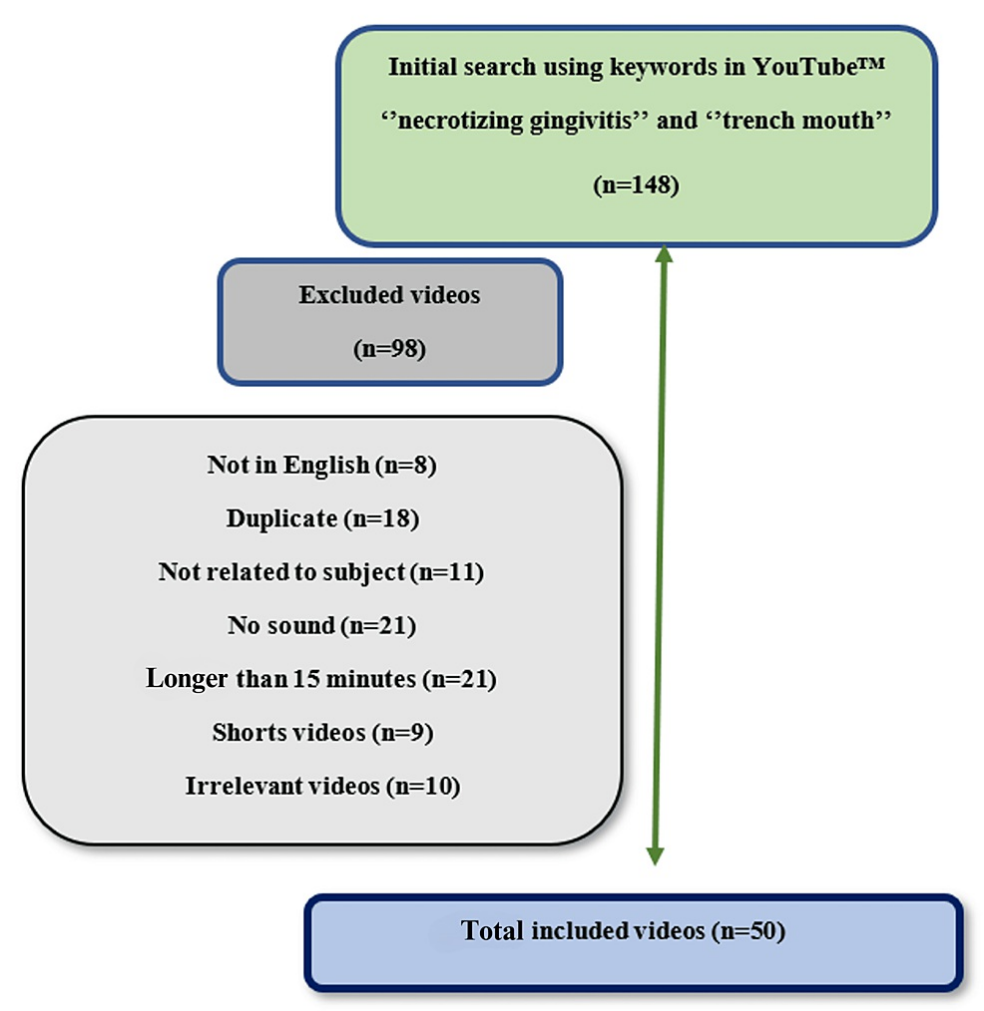
Flow diagram of the selection process Image Credit: Author

Initially, all 148 videos were carefully reviewed and analyzed. After excluding videos that were (a) not in English, (b) duplicates, (c) irrelevant, (d) silent, (e) short, and (f) irrelevant, our final sample consisted of 50 unique English-language videos, which formed the basis of our research and subsequent findings (Figure 1). All videos were watched by two independent periodontologists (Ş.Ç.B and O.K). Dr. ŞÇB has been a faculty member at the Department of Periodontology, Faculty of Dentistry, University of Health Sciences, and is known for her clinical experience and expertise in research. Dr. OK has been working in the Department of Periodontology of the same university, has received training on NG from many faculty members, and has read many books and articles on this subject. Both researchers adhered to the principles of impartiality and objectivity during the study process. The researchers did not declare any conflicts of interest that may have influenced the results of the study.

There was a break of at least three minutes between two consecutive videos to allow the investigators to concentrate. Videos were categorized according to the source of upload (healthcare professionals (periodontologists, dentists), hospital/university/dental clinics, commercial, layperson, other (TV channels, agencies), and the purpose of the videos (testimonial or educational).

For each video, views, upload date, comments, likes/dislikes, country of origin, and duration in minutes were recorded; however, the researcher who watched the video did not look at the number of comments or number of likes/dislikes before finishing watching the video so that the evaluation is not biased. Disputes among researchers regarding the evaluation of some videos were resolved by discussing the issues and reviewing the literature. Viewer interactions were calculated based on the interaction index (number of likes − number of dislikes/total number of views × 100%) and the viewing rate (total number of views/number of days since upload × 100%).

Video content was analyzed according to the topic domain that was either mentioned or not mentioned in the videos (Table 1). The videos scored from 0 to 7 points; those scored from 0 to 3 were classified as low-content and 4 to 7 as high-content.

**Table 1:** Topic domains analyzed in YouTube™ videos related to NUG-trench mouth NUG: necrotizing ulcerative gingivitis For each criterion, 1 point was given if mentioned in the video; 7 points possible: 0 to 3 = low-content, 4 to 7 = high-content

		Score
1	Description	1
2	Risk factors/etiologic factors	1
3	Pathophysiology	1
4	Clinical signs and symptoms	1
5	Treatment approach	1
6	Complications	1
7	Prevention	1
	Total score	7

Videos were reviewed for comprehensibility, applicability, reliability, and quality using standardized tools such as the Global Quality Scale (GQS) [15] and the Video Information Quality Index (VIQI) [16]. These tools were chosen for their ability to assess the quality of health information in videos using a standardized method.

A 5-point Likert scale GQS (1 point for very low quality, 2 points for limited use and low quality, 3 points for moderate quality, 4 points for good quality, and 5 points for excellent quality) was used to measure the flow, overall quality, and usefulness of the videos for patients [15]. The overall audiovisual quality was assessed by the VIQI using a 5-point Likert scale (1 = poor quality and poor flow, 2 = generally poor quality and poor flow, 3 = moderate quality and suboptimal flow, 4 = good quality and generally good flow, and 5 = excellent quality and flow) to assess parameters such as flow of information, information accuracy, quality (including animation, use of still images, video subtitles, interviews with individuals in the community, and report summaries), and precision (agreement between video title and content) [16].

Reliability and validity

In this study, reliability was evaluated using various methods to ensure the consistency and reliability of our findings. First, the test-retest method was employed by measuring the same variables by different researchers, and the level of agreement between the measurements was determined. The internal consistency of the measurement tools utilized in our study was assessed with Cronbach's alpha coefficient, and the results were found to be acceptable in terms of reliability. Additionally, interrater reliability was examined to ensure consistency in the data interpretation process among the researchers participating in the study. Any discrepancies or disagreements in the data interpretation process were resolved through discussions and compromises within the research team. Furthermore, standardized data collection procedures were implemented, and validated measurement tools were preferred to enhance reliability in the data collection process. Rigorous quality control measures were put in place at each stage of data collection and analysis to address potential biases. For instance, researchers underwent training and calibration sessions to minimize subjective biases and ensure uniformity in data collection procedures.

A test-retest reliability analysis was conducted to determine whether the values of the same variables re-measured by different individuals were similar. The similarity between the initial and repeated measurements was examined using Pearson's Chi-Square correlation, Spearman rank difference correlation, and the kappa statistic. The correlation coefficients between the measurements exceeded the minimum value of 0.70, and the kappa statistic was above 0.60. Given that the p-values were less than 0.05 and statistically significant, positive, and high-level relationships were identified. Consequently, the measurements were deemed to be stable and consistent.

Numerous steps were taken to ensure the validity of our findings. Initially, established and validated measurement tools and procedures were employed to guarantee the accuracy and consistency of the data collection process. Additionally, an extensive review of relevant literature was conducted to support the construct validity of our research variables and ensure alignment with existing theoretical frameworks. Moreover, a diverse range of data collection and analysis methods were utilized to bolster our findings and enhance the robustness of our conclusions. Any factors that could potentially compromise validity, such as confounding variables or errors in measurement, were meticulously assessed and analyzed in the interpretation of our results.

Statistical analysis

Descriptive statistics, including the number, percentage, mean, standard deviation, median, minimum, and maximum values, were computed to summarize the characteristics of the study sample. The Shapiro-Wilk test was conducted to assess the normality of the data distribution. This test helps determine whether the data follow a normal distribution, which is a key assumption for many statistical analyses. The choice of statistical tests was based on the distributional characteristics of the data and the research objectives. For example, when the data did not meet the assumption of normality, non-parametric tests such as the Mann-Whitney U test for comparing two independent groups and the Kruskal-Wallis test for comparing three or more groups were utilized. In instances where significant differences were observed among multiple groups, the post-hoc Bonferroni test was employed to identify specific group differences. This correction method helps mitigate the risk of Type I errors that may arise from conducting multiple comparisons. To explore relationships between variables, different correlation coefficients were used, depending on the nature of the variables. Kendall's Tau correlation coefficient was employed to assess the relationship between one continuous variable and one categorical variable, while the Spearman correlation coefficient was used to examine associations between two continuous variables. The Pearson Chi-Square test was utilized to examine the relationship between categorical variables when the sample size assumption (expected value > 5) was met. Alternatively, Fisher's exact test was applied when this assumption was not satisfied. All statistical analyses were performed using SPSS Statistics version 25.0 (IBM Corp. Released 2017. IBM SPSS Statistics for Windows, Version 25.0. Armonk, NY: IBM Corp.).

## Results

This study evaluated 50 YouTube™ videos. Table 2 shows the features of the analyzed videos, and Table 3 shows the characteristics of the analyzed videos. Table 4 presents the distribution of the countries where the videos were uploaded. Descriptive statistics for total content scores (TCS) are given in Table 5. The least mentioned topic in the video analysis was how to prevent NG (26%), while the most mentioned topic was the clinical signs/symptoms of NG (86%) (Table 5).

**Table 2:** Video features Min: minimum, Max: maximum, TCS: total content score, VIQI: video information and quality index

	Min	Max	Mean	Standard deviation	Median
Number of views	12.00	64565.00	3974.28	11520.19	407.00
Duration in seconds	33.00	895.00	363.50	219.77	365.00
Days since upload	59.00	5276.00	1450.12	1248.48	1125.00
Number of comments	0.00	45.00	3.98	9.07	0.00
Number of likes	0.00	1305.00	59.56	193.99	7.00
Viewing rate	0.01	25.02	2.40	4.77	0.59
Interaction index	0.00	0.11	0.02	0.02	0.02
TCS	0.00	7.00	4.16	1.93	5.00
VIQI	4.00	19.00	13.10	3.92	15.00

**Table 3:** Video characteristics GQS: global quality score

		n	%
Source of upload	Healthcare professionals	28	56
	Hospital/university	5	10
	Layperson	4	8
	Other	13	26
Video type	Educational	49	98
	Testimonial	1	2
GQS	Score 1	5	10
	Score 2	9	18
	Score 3	10	20
	Score 4	23	46
	Score 5	3	6

**Table 4:** Distribution of countries where the videos were uploaded

Countries	n	%
Canada	4	8.0
Egypt	1	2.0
India	23	46.0
Malaysia	2	4.0
Nepal	1	2.0
Pakistan	1	2.0
South Korea	1	2.0
UK	2	4.0
USA	15	30.0

**Table 5:** Descriptive statistics of TCS TCS: total content score

		n	%
Content 1	No	9	18
	Yes	41	82
Content 2	No	14	28
	Yes	36	72
Content 3	No	25	50
	Yes	25	50
Content 4	No	7	14
	Yes	43	86
Content 5	No	17	34
	Yes	33	66
Content 6	No	33	66
	Yes	17	34
Content 7	No	37	74
	Yes	13	26
TCS	Low content	16	32
	High content	34	68

In the comparison of video characteristics according to TCS, statistically significant differences were found between video duration, time since upload, and VIQI scores (p<0.05). The video duration and VIQI scores of HC videos were higher than those of LC videos. The time elapsed after uploading LC videos is longer than the time elapsed after uploading HC videos. Statistically significant differences were not obtained between the TCS and the number of views, number of comments, number of likes, viewing rate, and interaction index (p>0.05) (Table 6). According to the analysis of the relationships between TCS and GQS, as GQS increased, the TCS scores also increased (p<0.05). Statistically significant relationships were not obtained between the TCS and the source of upload or video type (Table 7).

**Table 6:** Distribution and comparison of video characteristics according to TCS *p<0.05, **independent sample t-test Min: minimum, Max: maximum, TCS: total content score, VIQI: video information and quality index

		Min-Max	Mean±standard deviation (median)	Test statistics	p-value
Number of views	Low content	16-64565	5093.63±15956.2 (346.5)	258	0.771
	High content	12-50516	3447.53±8968.86 (442.5)		
Duration in seconds	Low content	33-549	189.88±156.71 (118.5)	75	<0.001*
	High content	148-895	445.21±197.63 (422.5)		
Days since upload	Low content	148-5276	2447.31±1704.99 (2681)	3.364**	0.044*
	High content	59-2214	980.85±532.97 (1054.5)		
Number of comments	Low content	0-13	1.38±3.28 (0)	212.5	0.177
	High content	0-45	5.21±10.6 (0.5)		
Number of likes	Low content	0-108	15.69±26.56 (6)	224.5	0.292
	High content	0-1305	80.21±232.77 (8)		
Viewing rate	Low content	0.01-14.12	1.38±3.43 (0.51)	204	0.157
	High content	0.02-25.02	2.88±5.26 (0.97)		
Interaction index	Low content	0-0.07	0.02±0.02 (0.01)	204	0.156
	High content	0-0.11	0.02±0.02 (0.02)		
VIQI	Low content	4-13	8.50±2.99 (8)	15	<0.001*
	High content	11-19	15.26±1.93 (16)		

**Table 7:** Distribution of TCS by video characteristics and their relationships *p<0.05 TCS: total content score, GQS: global quality score, VIQI: video information and quality index

TCS		Low content		High content			
		n	%	n	%	Test statistics	p-value
Source of upload	Healthcare professionals	7	43.8	21	61.8	4.25	0.203
	Hospital/university	1	6.3	4	11.8		
	Layperson	3	18.8	1	2.9		
	Other	5	31.3	8	23.5		
Video type	Educational	15	93.8	34	100	-	0.32
	Testimonial	1	6.3	0	0		
GQS	Score 1	5	31.3	0	0	43.154	<0.001*
	Score 2	9	56.3	0	0		
	Score 3	2	12.5	8	23.5		
	Score 4	0	0	23	67.6		
	Score 5	0	0	3	8.8		

The correlation coefficients calculated between VIQI scores and video duration, time since upload, viewing rate, and interaction index showed statistically significant, positive-negative, and moderate relationships (p<0.05). The same relationship was observed between TCS and video duration, time since upload, and VIQI. The correlation coefficients calculated between GQS scores and video duration, time since upload, viewing rate, and VIQI showed statistically significant, positive-negative, and low-medium-high level relationships (p<0.05) (Table 8).

**Table 8:** Relationships between features of YouTube™ videos and VIQI, TCS, and GQS *p<0.05 r: correlation coefficient, †: Kendal's Tau correlation, TCS: total content score, GQS: global quality score, VIQI: video information and quality index

		VIQI	TCS†	GQS†
Number of views	r	0.143	0.034	0.065
	p	0.322	0.771	0.551
Duration in seconds	r	0.313	0.483	0.302
	p	0.027*	<0.001*	0.005*
Days since upload	r	-0.463	-0.3	-0.396
	p	0.001*	0.011*	<0.001*
Number of comments	r	0.215	0.175	0.12
	p	0.133	0.177	0.312
Number of likes	r	0.265	0.127	0.169
	p	0.063	0.292	0.127
Viewing Rate	r	0.378	0.167	0.26
	p	0.007*	0.157	0.016*
Interaction Index	r	0.306	0,169	0.212
	p	0.031*	0.156	0.053
VIQI	r	1	0.669	0.868
	p	-	<0.001*	<0.001*

## Discussion

According to consumer-based assessments, individuals tend to prefer social media platforms over scientific ones when seeking information on various topics. Consequently, there is a growing need for social media platforms like YouTube™ to be overseen or managed by professionals to ensure the dissemination of accurate information to users [17]. YouTube™ stands out among social media platforms due to its provision of rich visual content and easy accessibility to information, making it a popular choice among patients. However, concerns have been raised because YouTube™ videos are easily shareable and the content uploaded is not standardized [18]. Therefore, clinicians should bear in mind that, despite the accuracy of video content, viewers may interpret it differently [19].

While evaluations of YouTube™ videos have been conducted on various dentistry-related topics such as dentine hypersensitivity, vital pulp capping, gummy smile surgical treatment, and sinus lifting surgery [17,20-22], there has been no study evaluating the information quality of YouTube™ videos on NG. Despite using appropriate search terms, both physicians and patients encounter difficulties in obtaining relevant information due to redundant content. Out of the 148 videos identified in the search for "necrotizing gingivitis" and "trench mouth," 98 were found to be irrelevant, lacked audio, were not in English, or were duplicated. Following the exclusion of these videos, the remaining 50 YouTube™ videos were analyzed for content quality. NG is particularly prevalent among young individuals, severely malnourished children, and adults with HIV infection [23]. Treatment guidelines published by the American Academy of Periodontology stress that NG treatment necessitates a multifaceted approach, combining personal plaque control and professional debridement to reduce oral bacteria for prompt control. Without adequate oral hygiene care, relapses are likely to occur, or more serious complications may arise. Additionally, physicians may mistake the diagnosis of NG for many bacterial and viral conditions, prompting many patients and physicians to seek information on social media [24]. The number of views of the 50 videos included in this study ranged from 12 to 64,565. This demonstrates that videos' view counts can reach high numbers and potentially mislead many people when the content is insufficient.

In this study, a significant relationship was found between TCS, video duration, time since upload, and VIQI scores (p<0.05). HC videos had higher total VIQI scores and longer video durations than LC videos. The significant difference in video durations indicated that increasing video duration was also indicative of increasing TCS. Our results are consistent with the positive relationship between TCS, VIQI, and video duration as observed in the studies by Hatipoğlu et al. [25] and Tamošiūnaitė et al. [26], while conflicting with the results of Topbaş et al. [21]. This inequality between studies could be explained by the presence of different TCS criteria. Videos should be only long enough to explain the content and key points of the topic concisely without repetition [27]. Therefore, videos over 15 minutes were excluded from our study.

In our study, the time since the upload of LC videos was higher than the time since the upload of HC videos. This situation may arise from advancements in technology, improved production techniques, and feedback on shortcomings from previous videos, all of which contribute to developments in the content creation process. However, it should be kept in mind that these variables may be manipulated by factors, including the number of followers and advertisements.

In the current study, the TCS score did not show a statistically significant difference based on the source of YouTube™ videos. Our results indicated that over half of the analyzed videos were published by healthcare professionals and hospitals. While the results of Meşeli et al. [17] and Sadry and Büyükbaşaran's [27] studies are consistent with our findings regarding the video source, Şen et al. [28] and Atagün et al. [20] studies found that the source of videos influenced the TCS score. This discrepancy could be explained by the fact that the uploader's content-related knowledge did not significantly affect the TCS scores. According to this study, the rich-content video groups had higher GQS scores. Because accurate and balanced information is more beneficial for patients. These findings are consistent with the findings of Atagün et al. [20] and Topbaş et al. [21].

According to our study, there was a positive correlation between VIQI, TCS, GQS, viewing rate, and video duration at different strengths and a negative correlation between them and the time since upload. As GQS scores increased, VIQI scores, which measure video credibility, the richness of content, information flow, accuracy, quality, and precision, increased. Information flow, accuracy, use of elements (such as images and captures), animations, and appropriate title selection improve the quality of videos. Videos that contain a greater amount of diverse and accurate content are more beneficial to patients, which explains the correlation between the TCS and GQS points. Similarly, the correlation between VIQI and TCS is also considered a result of YouTube™ users following the developing technology. Videos with good information are more valuable for patients. In addition, when conveying scientific content through videos, the adequacy of the information increases the quality, usability, and usefulness of the video for patients. In this respect, our findings are consistent with those of Topbaş et al. [21] and Hatipoglu et al. [25].

No correlation was found between likes, dislikes, the number of comments, and VIQI, TCS, and GQS. Şen et al. [28] had similar findings to ours on this issue; Nason et al. [19] and Bulut et al. [29] found that the quality, reliability, and understandability of the videos were related to viewing and liking rates. It can be said that patients watch at high rates and can interact with other users through comments, likes, and dislikes. However, it is important to note that the popularity and visibility of online videos are not always linked to the quality and comprehensiveness of the videos. Because the majority of patients who watch the videos do not question or judge the quality or content of the videos. Additionally, parameters such as subscribers, likes, dislikes, or the number of comments are potentially subjective and manipulable YouTube™ metrics. They are not determining factors for video usability.

We must be cautious about the external validity of our findings, given the time specificity of when our data was collected. As the content and visibility of YouTube™ videos may change over time, it is important to note that the results of our study may only be valid for a specific time period. Therefore, when assessing the external validity of YouTube™ videos on NG, we need to consider the time period in which data collection took place, and it is also important to acknowledge how the outcomes of assessments made in different time periods may vary in future research.

Limitations and future directions

This study has several limitations, including its cross-sectional design and analysis of only a subset of a comprehensive data pool. The YouTube™ platform is dynamic, constantly receiving new data and allowing old data to be deleted and/or modified. While this study analyzed only 50 videos during a specific period, the vast content volume on YouTube™ is evident; with 48 hours of video uploaded every minute, significant amounts of content are added daily. It is important to acknowledge that since the data collection and evaluation occurred, there may have been significant changes in the TCS, GQS, and VIQI scores of the available information on the platform. Additionally, video optimization and analytics can influence the search results and ranking of the same search item for different users. Temporal variations in research areas and search results contribute to natural variability, making it difficult to replicate studies. No specific guidelines were used to calculate the usability score of the videos. Contents deemed important for usability scoring were identified by the researchers and subjectively evaluated. Furthermore, the use of subjective tools like GQS and VIQI to assess videos relies on observer interpretation.

Another aspect that should be considered in our study is the potential selection bias resulting from our reliance on specific keywords and a single language for conducting YouTube™ video searches. By doing so, we may inadvertently overlook a broader range of educational content that could be relevant to our inquiry. Further research could be conducted using different terms and evaluation scales in this regard.

Future studies can contribute to the existing literature by exploring alternative keywords or search criteria. Analyzing different keywords could provide a more comprehensive understanding of the video landscape and potentially uncover additional gaps in information coverage. Additionally, focusing on specific aspects such as prevention, treatment options, or patient experiences may offer unique insights and complement existing research.

## Conclusions

The main finding of the study indicates that the majority of YouTube™ videos on NG are of high quality and positively rated in terms of content, supported by above-average GQS and VIQI scores and 68% HC videos. However, some videos contain misleading or insufficient information. Additionally, the fact that only 34% of the analyzed videos (n=50) met our inclusion criteria emphasizes the need for improved access to accurate information on critical health issues such as NG. Consequently, respected health organizations such as the European Federation of Periodontology or the American Academy of Periodontology should play a more active role in producing videos related to NG. Although these organizations were not present in the analyzed videos, their valuable expertise and authoritative content could significantly contribute to increasing awareness. Including videos from these organizations could enhance the reliability and credibility of the information available on YouTube™.

Since social media is used not only for NG but also for accessing health-related information in many other fields, government agencies and health organizations should work together to establish rules to take strict measures against those circulating unverified and unreliable information on platforms like YouTube™. Additionally, it's essential to emphasize the need for patients to be cautious when obtaining health information from digital platforms. Healthcare professionals can play a crucial role in guiding patients to reliable sources and assisting them in making informed decisions based on evidence.
